# RESILIENCE THROUGH ELDERSHIP

**DOI:** 10.1093/geroni/igae098.1728

**Published:** 2024-12-31

**Authors:** Lena Thompson, Steffi Kim, Jordan Lewis

**Affiliations:** University of Minnesota Duluth, Duluth, Minnesota, United States; University of Alaska, Anchorage, Alaska, United States; University of Alaska Fairbanks, College of Indigenous Studies, Fairbanks, Alaska, United States

## Abstract

Alaska Native (AN) Elders face significant challenges aging in place, yet Elders find joy and connection from living in their villages and often choose to remain in their villages as they age. As keepers of wisdom and knowledge bearers, AN Elders serve as guides and collaborators in overcoming challenges on individual, community, and at policy-levels. This study collects stories and strategies of AN Elder resiliency fostered through leadership, community engagement, and cultural practices in Southeast and Interior Alaska. This study comes from a parent study exploring meanings of and strategies for “aging in a good way” among Alaska Native Elders. In-person semi-structured interviews were conducted with 72 Elders from 11 communities in Southeast and Interior Alaska. Recordings were transcribed, de-identified, and coded thematically. Elders described navigating life-long challenges, related to experiences of racism, generational trauma, and generational changes. Elders described ways that they address these life challenges, including practices and beliefs rooted in traditional teachings, gathering, hunting, and living their “native way of life.” Notably, Elders felt that it was their duty to pass knowledge on to younger generations to address their own life challenges, incorporate and continue traditional practices, and save them hardships experienced by Elders. The concept of Eldership is vital to individual, family, and community wellbeing promoting bidirectional and mutually beneficial relations within youth, communities, tribes, and organizations. Through Eldership, AN Elders provide important guidance in managing challenges which can support Tribal governments and other service systems as they determine effective strategies to support AN communities.

